# Device-based Optimization of Cardiac Resynchronization—One Size Does Not Fit All

**DOI:** 10.19102/icrm.2022.130307

**Published:** 2022-03-15

**Authors:** Wasim Rashid, Asim Kichloo, Khalil Kanjwal

**Affiliations:** ^1^Department of Cardiology, Sheri Kashmir Institute of Medical Sciences, Srinagar, India; ^2^Department of Internal Medicine, Central Michigan University, Mt Pleasant, MI, USA; ^3^Department of Cardiology, McLaren Greater Lansing Hospital, Lansing, MI, USA

**Keywords:** Cardiac resynchronization therapy, cardiomyopathy, defibrillator, right bundle branch block, Sync-AV

## Abstract

We report on a 72-year-old female patient who was sent to our clinic for evaluation of a biventricular intracardiac defibrillator (BIV-ICD). The patient was diagnosed with ischemic cardiomyopathy and showed a persistently low ejection fraction in the range of 20%–25% with New York Heart Association class III heart failure symptoms despite being on guideline-directed medical therapy, including a β-blocker and a combination of sacubitril and valsartan, for >3 months. In addition, the patient had underlying right bundle branch block (RBBB) with a QRS duration of 160 ms. The device was programmed with a Sync-AV algorithm on with nominal settings (delta of −50 ms). The thresholds and lead impedances were acceptable. Electrocardiography was performed in the postoperative period, showing persistent RBBB similar to the baseline electrocardiogram without much QRS narrowing. In this report, we discuss the mechanism and troubleshooting of this problem.

A 72-year-old woman was sent to our clinic for the evaluation of biventricular-intracardiac defibrillator (BIV-ICD). The patient was diagnosed with ischemic cardiomyopathy and was having persistently low ejection fraction in the range of 20%–25% with New York Heart Association class III heart failure symptoms despite being on guidelines-directed medical therapy, including a β-blocker and sacubitril–valsartan combination, for >3 months. In addition, the patient had underlying right bundle branch block (RBBB) with a QRS duration (QRSd) of 160 ms **(Figure 1)**. In view of her persistent cardiomyopathy and QRSd of >150 ms, it was decided to implant a BIV-ICD. The left ventricular (LV) lead was implanted in the mid-lateral branch. The device was programmed with a Sync-AV algorithm (Abbott, Chicago, IL, USA) on with nominal settings (delta of −50 ms). The thresholds and lead impedances were acceptable. An electrocardiogram (ECG) was performed in the postoperative period, which showed persistent RBBB **(Figure 2)** similar to the baseline electrocardiogram (ECG) without much QRS narrowing. The device interrogation was repeated, and all the lead parameters were stable with acceptable thresholds. What is the reason for persistent wide QRS and lack of desired QRS narrowing in this patient with baseline RBBB?

## Understanding the Sync-AV algorithm and its limitations

Cardiac resynchronization therapy (CRT) is an established evidence-based therapy in patients with systolic heart failure and wide QRS.^1^ However, not all patients respond to this therapy, and such patients are labeled as non-responders. Almost one-third of patients with advanced heart failure who undergo CRT implantation are non-responders.^2^ Furthermore, response to CRT is a continuum rather than a binary outcome.^3^ Hence, there is a lot of interest in the optimization of CRT post-implant in non-responders as well as maximizing outcomes in those who are considered responders. Further, multiple studies have shown that paced ECG QRSd can be used as a surrogate for the effectiveness of resynchronization therapy as well as patient outcomes.^4^ A variety of post-implant issues besides patient selection may contribute to suboptimal responses. The most common reasons are non-left bundle branch block (LBBB), suboptimal lead location, and a narrower QRS complex. Post-implant device optimization predominantly focuses on the modification of atrioventricular (AV) and interventricular timing delays either manually or with the use of proprietary algorithms. The aim of optimizing these delays is to obtain a narrower paced QRS complex as a metric of electrical resynchronization. One of the proprietary algorithms for optimizing the AV delays (AVDs) is the Sync-AV algorithm (Abbott) that dynamically adjusts AVD to synchronize ventricular pacing with each beat’s intrinsic AV conduction.

Briefly, Sync-AV functions as follows. Every 256 beats, the algorithm automatically extends the paced and sensed AVD for 3 beats, during which it measures the intrinsic AV interval. With the default Sync-AV offset, 50 ms is subtracted from the measured intrinsic AV interval, and the result is applied as the paced AV interval for the following 255 beats (ie, Sync-AV paced AVD = intrinsic AV interval − 50 ms). The cycle repeats every 256 beats, thus permitting dynamic adjustment of the paced AV interval. The offset value may be reprogrammed across a wide range of offsets (10–120 ms).

The initial study of Sync-AV^5^ examined the effect of Sync-AV on paced QRSd in 73 patients and showed that, in already well-selected patients with LBBB and optimized LV lead position, patient-tailored BIV pacing adjusted to intrinsic AV timing using Sync-AV resulted in the narrowest QRSd. Further studies showed an improvement in acute hemodynamic measures and reverse remodeling on echocardiography.^6,7^ Also, Sync-AV fusion pacing provided the greatest improvement in electrical synchrony compared to conventional CRT and multipoint pacing as well as having a synergistic effect when superimposed on the latter.^6,8^ However, these studies were almost exclusively performed in patients with LBBB. Hence, current recommendations for using the Sync-AV algorithm include patients with (1) intact AV conduction PR, (2) PR <300 ms; (c) LBBB, (d) minimal ventricular ectopy, and (e) low atrial tachycardia/atrial fibrillation burden. It is intuitive to think that similar programming cannot lead to optimal electrical synchrony in both RBBB and LBBB patients as the activation patterns are different.

## Discussion

Although the American College of Cardiology/Heart Rhythm Society/European Society of Cardiology recommend CRT in patients with a QRSd of >150 ms, there is a paucity of data on the utility of CRT in patients with RBBB. Despite recommendations of CRT in patients with a QRSd of >150 ms, there are no clear-cut guidelines regarding the utility of CRT in patients with RBBB.^1^ In patients who receive CRT devices, post-implant optimization is routinely performed with an aim to achieve a narrower QRSd. One post-implant optimization analysis showed that suboptimal programming of AV timings was found to be contributory in as high as 47% of patients with a poor response to CRT.^9^ Early trials showed significant changes in acute hemodynamics (LVdp/dt max) with different AVDs, and AVD optimization using echo parameters showed a small benefit in clinical outcome.^10,11^ However, larger prospective studies did not replicate these results.^10,12^ This was thought to be due to the dynamic nature of the intrinsic AV intervals, dependent on heart rate, autonomic tone, and other factors, where “best” AVDs may vary significantly over time. This led to a search for automated algorithms to assess intrinsic AV times, and thus several proprietary algorithms for programming AVDs were developed. Several CRT studies demonstrated a link between acute QRS narrowing and long-term clinical response.^4,11,13^ Consequently, many studies have been done to optimize device programming post-implant by modification of the AVDs aimed at achieving a narrower QRSd.

**Figure 3:**
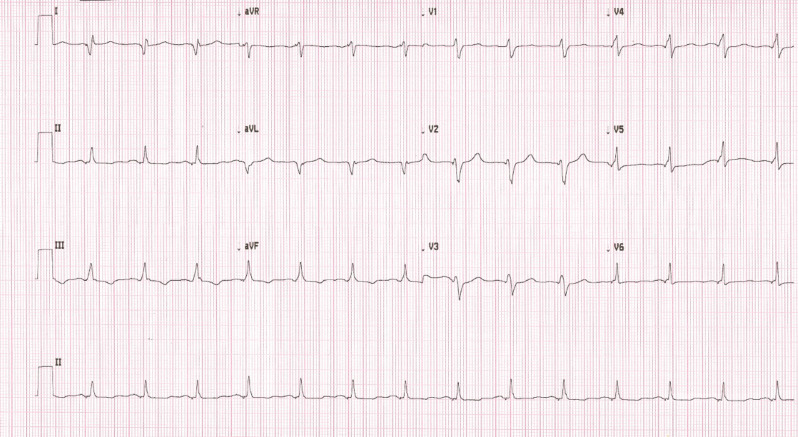
Electrocardiogram with Sync-AV off and fixed atrioventricular delays programmed.

In our case, Sync-AV fusion pacing stimulation resulted in a wider QRS than CRT with a nominal fixed AVD. After confirming that the thresholds were stable, the Sync-AV algorithm was turned off and manual optimization was performed with an AVD of 130–150 ms with an LV offset of 0 ms. This programming resulted in significant narrowing of QRS and disappearance of RBBB **(Figure 3)**. The patient was discharged home in a stable condition.

**Figure 4:**
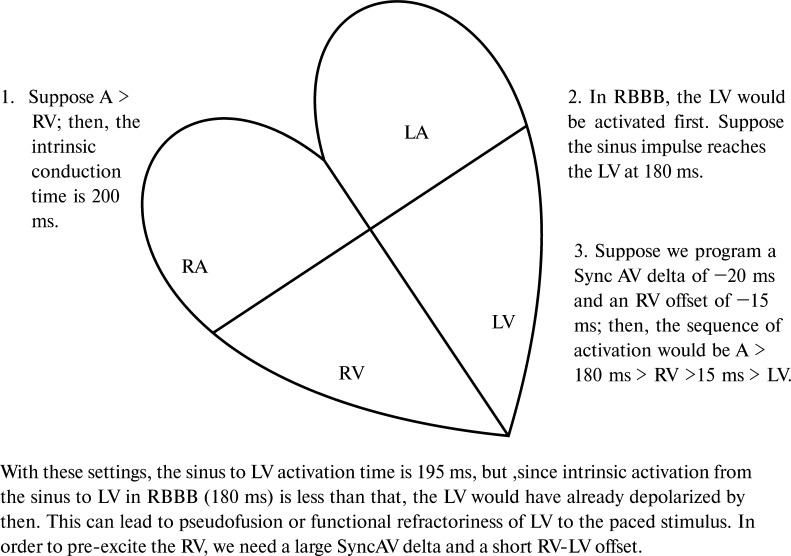
Image illustrating the consequences of programming default Sync-AV delta in the presence of right bundle branch block and the possible implications for device programming in this setting. *Abbreviations:* LA, left atrium; LBBB, left bundle branch block; LV, left ventricle; RA, right atrium; RBBB, right bundle branch block; RV, right ventricle.

The mechanism behind this phenomenon may be the inability to achieve triple wave-front fusion in RBBB with Sync-AV on. Using programming parameters originally tested for patients with LBBB may lead to pseudo-fusion between biventricular pacing and intrinsic beat in RBBB or pseudo-fusion with little contribution from the pacing stimulation. This has been depicted in **Figure 4**. As demonstrated in the figure, it would take a large Sync-AV delta and a short LV offset timing to achieve proper fusion pacing in patients with RBBB. Also, in the St. Jude devices, the sensing is performed by a right ventricular (RV) lead only. Thus, it is also possible that, with a longer AVD, the impulse reached the RV at the same time as the RV pacing artifact was delivered (pseudo-fusion). This could also lead to the functional loss of captures from the LV lead and, thus, no change in the QRS was noted post-implant with Sync-AV on.

Studies evaluating the utility of Sync-AV fusion pacing in patients with RBBB are sparse. In 1 study,^14^ the performance of Sync-AV was evaluated with multiple LV pacing sites in a broader cohort of 99 patients of RBBB, and nonspecific intraventricular conduction delay was observed in 5% and 32% of patients, respectively. The authors reported that the reduction in QRSd was not significantly influenced by the presence or absence of LBBB. In another study,^15^ 8 consecutive patients with RBBB at a single tertiary care center, who were implanted with a CRT device capable of biventricular fusion pacing using Sync-AV programming, were assessed and compared to a historical cohort of CRT patients without RBBB. With programming optimization, they could demonstrate that the reduction in QRSd with optimized Sync-AV observed in those with RBBB approached the level of that seen in patients with LBBB. In those with LBBB, the optimal Sync-AV offset was 30–50 ms in around 66%–80% of patients, and the majority of the remaining patients had an optimal offset of <30 ms. In contrast, it was found that in patients with RBBB, the optimal offset was around 90 ms in the vast majority of patients. This finding is key in considering the optimal negative AV hysteresis offset required to achieve fusion; given that the AVD is measured by the device using the RV lead, the presence of RBBB results in the delayed detection of ventricular activation (relative to surface ECG) and the need to program a more negative AV offset to achieve fusion. These considerations are similar to those depicted in **Figure 3**. However, the degree to which fusion can be achieved with fixed AVD adjustment in RBBB patients without a dynamic fusion pacing algorithm was not evaluated in this study. In our case a fixed nominal AVD led to better electrical synchrony with a narrower QRS on the ECG **(Figure 3)**. One of the limitations of this case is that, although the QRSd decreased with manual optimization, its long-term effects on patient outcomes are unknown.

## Conclusion

This case highlights that Sync-AV fusion pacing may not be ideal for patients with RBBB in achieving electrical synchrony. Larger comparative studies are needed to address the nuances of CRT programming and device-based optimization in patients with RBBB and other non-LBBB intraventricular conduction delays.

## Figures and Tables

**Figure 1:**
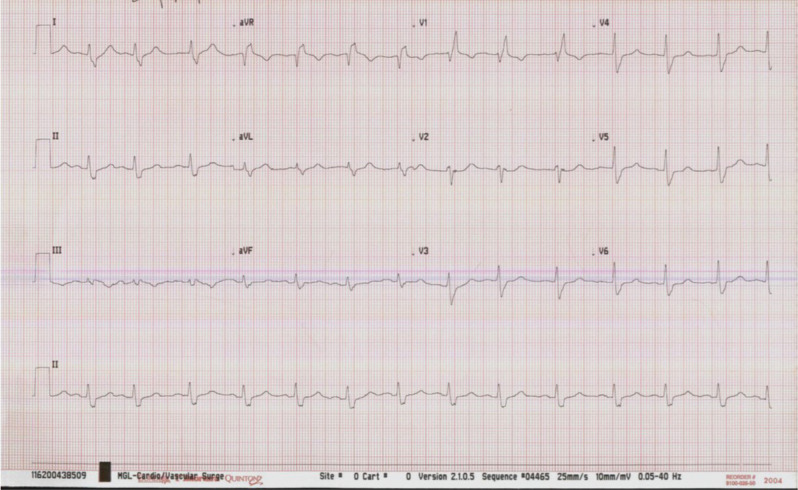
Baseline electrocardiogram before device implantation.

**Figure 2:**
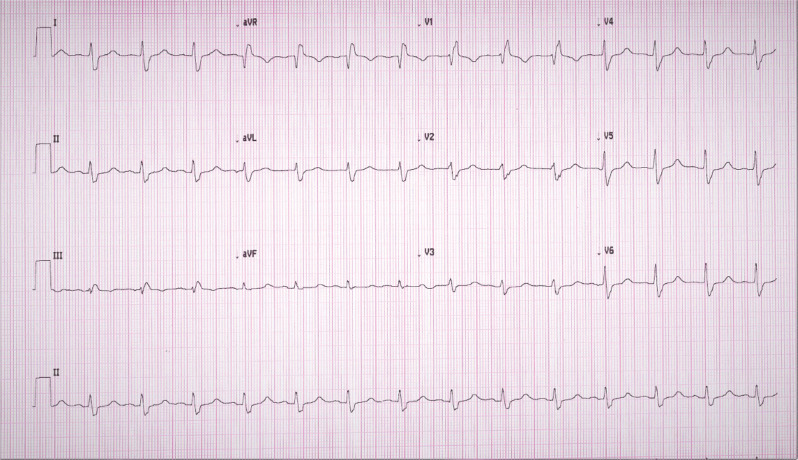
Immediate post-device implant electrocardiogram (Sync-AV on).
